# Epidemiology and genetic diversity of *Anaplasma ovis* in goats in Corsica, France

**DOI:** 10.1186/s13071-018-3269-7

**Published:** 2019-01-03

**Authors:** Alejandro Cabezas-Cruz, Mélanie Gallois, Mélanie Fontugne, Eléonore Allain, Myriam Denoual, Sara Moutailler, Elodie Devillers, Stephan Zientara, Marc Memmi, Alain Chauvin, Albert Agoulon, Muriel Vayssier-Taussat, Christophe Chartier

**Affiliations:** 1 UMR BIPAR, INRA, ANSES, Ecole Nationale Vétérinaire d’Alfort, Université Paris-Est, 94700 Maisons-Alfort, France; 2 Fédération Régionale des Groupements de Défense Sanitaire du Bétail de Corse (FRGDSB20), 20090 Ajaccio, France; 3 BIOEPAR, INRA, Oniris, 44307 Nantes, France; 4 UMR VIROLOGIE, INRA, Ecole Nationale Vétérinaire d’Alfort, ANSES, Université Paris-Est, 94700 Maisons-Alfort, France; 5 Laboratoire d’Analyses de Corse, site de Bastia, 20600 Bastia, France; 6 Département Santé Animale, INRA, 37380 Nouzilly, France

**Keywords:** *Anaplasma ovis*, Prevalence, Dairy goat, *Rhipicephalus bursa*, Corsica, France, Genetic diversity

## Abstract

**Background:**

*Anaplasma ovis* is a major cause of small ruminant anaplasmosis, a tick-borne disease mainly affecting small ruminants in tropical and subtropical regions of the world. Due to health and production problems in dairy goat flocks in Corsica, France, and the demonstration of *A. ovis* infection in some animals, an extensive survey was conducted in the island in spring 2016. The aim of the survey was to determine the prevalence and geographical distribution of *A. ovis* infections in goats and ticks as well as possible relationships with anaemia and other health indicators. In addition, the genetic diversity of *A. ovis* was evaluated.

**Methods:**

Blood and faecal samples were collected in 55 clinically healthy flocks (10 goats per flock) for *A. ovis* qPCR, haematocrit determination, paratuberculosis ELISA seropositivity and gastrointestinal nematode egg excretion quantification. Ticks were collected, identified and processed for *A. ovis* DNA detection.

**Results:**

A high prevalence of *A. ovis* DNA detection was found at the individual (52.0%) and flock levels (83.6%) with a within-flock prevalence ranging between 0–100%. *Rhipicephalus bursa* was the only tick species collected on goats (*n* = 355) and the detection rate of *A. ovis* DNA in ticks was 20.3%. *Anaplasma ovis* DNA prevalence was higher in flocks located at an altitude above 168 m, in goats of Corsican/crossbred breed and in goats > 3 years-old. No relationship was found between *A. ovis* DNA detection at the individual or flock level and haematocrit, paratuberculosis seropositivity or gastrointestinal parasites. Positive *A. ovis* goat samples were used for amplification of *gltA* and *msp4* genes for species confirmation and strain identification, respectively. Sequence and phylogenetic analysis of these genes confirmed the detection of *A. ovis* and allowed identification of six different strains of this pathogen (named Corsica 1-6 (COR1-6). While the *msp4* sequence of strain COR1 had 100% identity with strains previously reported, COR2 to 6 were found to be novel strains. The strain COR1 was the most represented, corresponding to 94.6% of the *msp4* sequences obtained.

**Conclusions:**

The results showed a relatively high genetic diversity of *A. ovis* associated with high bacterial prevalence in goats.

**Electronic supplementary material:**

The online version of this article (10.1186/s13071-018-3269-7) contains supplementary material, which is available to authorized users.

## Background

Bacteria of the genus *Anaplasma* (Rickettsiales: *Anaplasmataceae*) are obligate intracellular microorganisms including important human (e.g. *A. phagocytophilum*), livestock (e.g. *A. phagocytophilum*, *A. marginale* and *A. ovis*) and pet (e.g. *A. platys*) pathogens. These pathogens are mainly transmitted by tick bites, though other modes of transmission have been reported such as hematophagous insect bites and exposure to blood-contaminated fomites [1, 2]. *Anaplasma ovis* is distributed worldwide and is considered as the most frequent cause of small ruminant anaplasmosis but seems to be less pathogenic than other *Anaplasma* species, causing only subclinical infections with a low grade fever [3]. The bacterium may cause a persistent infection and, in some instances, clinical cases related to haemolytic anaemia may be seen with pallor and icterus, but without haemoglobinuria. The general clinical signs of *A. ovis* infection include fever, fatigue, anorexia, decrease in milk production and abortion but with a low mortality rate [4]. Outbreaks of acute disease are rare and occur mostly under major stress conditions (e.g. hot weather, undernutrition, low body condition score, vaccination, heavy tick infestation, long-distance transportation and animal movement) and more frequently in goats than in sheep [1, 5]. Additionally, as for other *Anaplasma* spp., *A. ovis* infection might predispose to other microbial or parasite infections resulting in exacerbated clinical signs and eventually death [6]. Genetic diversity of tick-borne bacteria resulting in novel strains can be associated with changes in pathogenicity, virulence, shift in host range, prevalence and enhancement of the transmission [7–11]. High genetic diversity of *A. marginale* was associated with an outbreak of bovine anaplasmosis in an endemic area in Mexico [12], and low genetic diversity in the *msp4* gene was associated with low prevalence of *A. ovis* in 12 provinces in China [13]. Assessment of genetic variability and strain diversity might therefore be crucial to understanding the epidemiology of anaplasmosis and to implementing control measures.

The economic significance of *A. ovis* outbreaks may be important in countries where animal stocks consist mainly of sheep and goats [14, 15]. However, subclinical disease and the natural resistance acquired by autochthonous *A. ovis* strains in endemic countries could make it difficult to evaluate the real economic impact of *A. ovis* infection [16].

Several wild animal species can be infected by *A. ovis* which further complicates the epidemiological cycle of this bacterium [17, 18]. There are numerous reports of high *A. ovis* prevalence in different regions of the world and values of up to 40% in *A. ovis* prevalence are commonly reported in the literature [19–21]. Corsica is an island where small ruminant breeding represents an important economic activity. Sheep and goat production is the third most important agricultural activity of the island [22]. Goat breeders produce 37% of the milk of Corsica [23]. In September 2013, an outbreak of bluetongue virus genotype 1 (BTV-1) was reported in sheep and goat in southern Corsica and the virus spread across the island in early 2014 [24]. Meanwhile, health and production problems were declared by several dairy goat farmers throughout the island during the spring of 2014, including emaciation and a drop in (or even drying up of) milk production, problems that did not seem to be due to BTV1. A preliminary study was then performed by the Regional Livestock Health Association (FRGDSB20) in five affected flocks through individual blood, milk and faecal sampling, as well as a few necropsies (Stephan Zientara and Mélanie Gallois, unpublished). The main investigated pathogens were gastrointestinal nematodes (GIN) and *Fasciola* (quantitative coproscopy), BTV (rt-RT-PCR), *Mycoplasma* spp. (bacteriological culture) and different *Anaplasma* species (qPCR) among others. The main results indicated high levels of GIN egg excretion and a high prevalence of *A. ovis* infection together with gross lesions suggestive of paratuberculosis (PTB) for five of the 11 necropsies. Due to the controversial clinical impact of *A. ovis* infection, it was not clear whether *A. ovis* could be responsible, alone or in association, for the described health problems in the studied goat herds. As a result, the FRGDSB20 decided to perform an extensive prevalence survey on *A. ovis* infection in dairy goats in Corsica.

Thus, the objectives of the present survey were first to determine the prevalence of *A. ovis* DNA in goats and ticks and their geographical distribution in dairy goats in Corsica; secondly to determine the relationship of *A. ovis* infection to other health indicators, namely anaemia, GIN egg excretion and PTB seropositivity; and lastly to investigate the genetic diversity of *A. ovis* of goat origin.

## Methods

### Study design

**Fig. 1:**
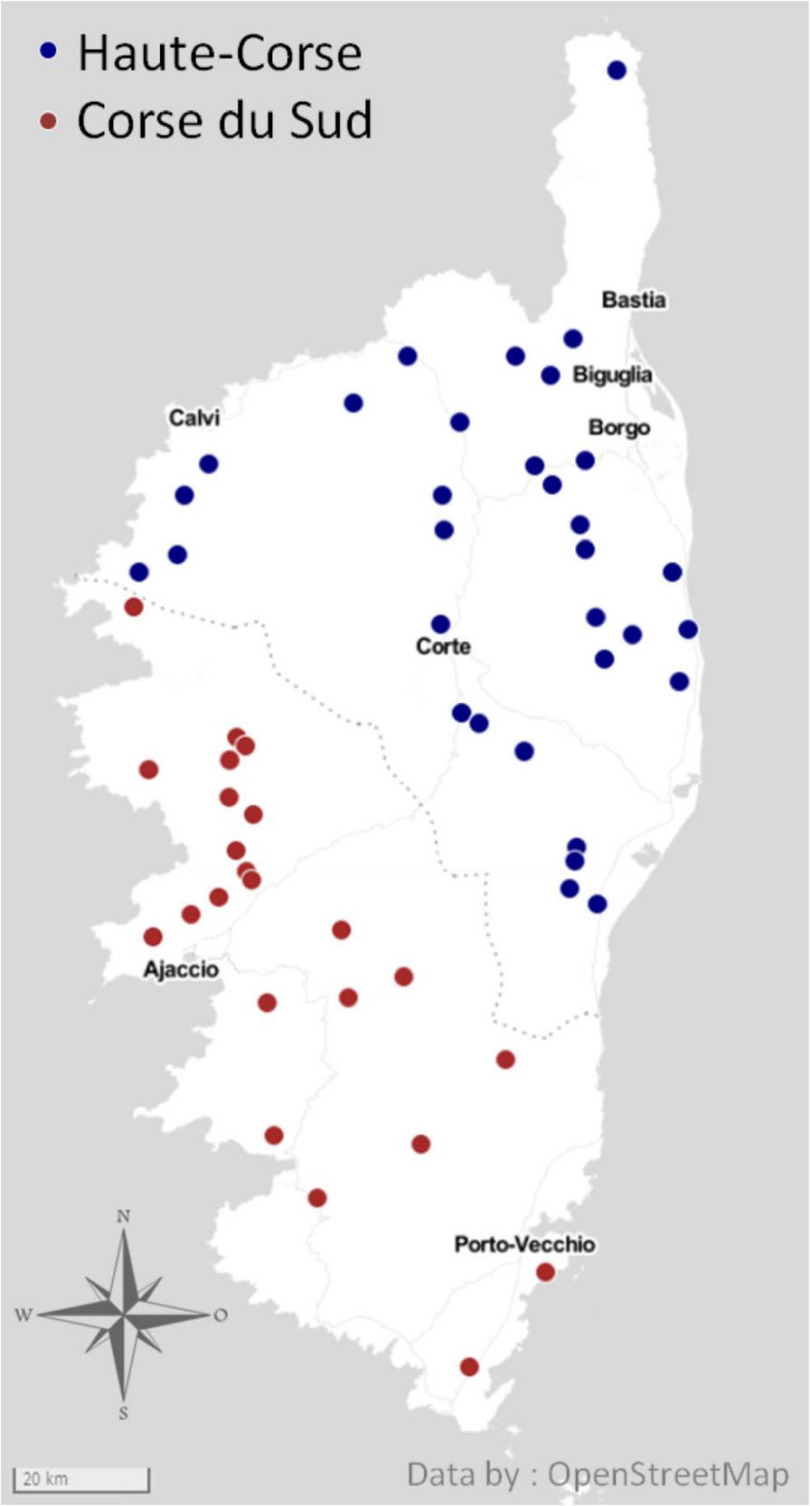
Map of Corsica showing the location of the 55 sampled goat flocks

Goat farms participating in the present survey were selected on a convenient basis according to geographical location to represent the breadth of the island. Fifty-five breeders volunteered to participate in this study: 32 from Haute-Corse and 23 from Corse-du-Sud counties (Fig. 1). No particular health problems were reported in the selected flocks at the time of the survey.

### Sample collection

The field survey was performed between April and June 2016. This period of time was chosen as it fitted with the maximal level of tick infestation of ruminants in Corsica [25]. Ten goats were randomly selected from each of the 55 herds, corresponding to 550 samples in total. To minimize a possible influence of age on the final within-flock outcome, the age of sampled goats was between 2.5–3.5 years. Blood samples were collected from the jugular vein of goats in plain tubes containing EDTA. Individual faecal samples were collected from rectum.

### Tick sample collection and identification

Ticks were collected from each goat mainly around the perineum and udder. All ticks were identified under a dissecting microscope according to the keys provided in [26]. All collected ticks were surface-sterilized, conserved in 70% alcohol and further processed for molecular analysis as previously described [27].

### Laboratory exams

Hematocrit (Ht, as %) was determined with Hemogold® automat (Kitvia SAS, Labathe-Inard, France), and the cut-off for anemia was defined as ≤ 24%. For each farm, an equal part of the 10 individual faeces (2 g per goat) was mixed to give a pooled faecal sample [28]. The faecal egg count (FEC) of gastrointestinal nematode eggs (expressed as eggs per gram of faeces, epg) was assessed from the pooled sample with the McMaster technique according to Raynaud [29]. FEC can be interpreted as follows: low excretion, < 500 epg; moderate excretion, 500–2000 epg; high excretion > 2000 epg [30]. Individual seroprevalence to paratuberculosis (PTB) was determined using an ELISA assay (IDEXX Paratuberculosis Screening Ab test®, IDEXX France SARL, Saint-Denis, France) following the manufacturer’s instructions. Results were expressed as positive or negative. Doubtful results were considered as negative.

### Molecular detection of *Anaplasma ovis*

#### DNA extraction

DNA was extracted from 200 μl of blood homogenate using DNeasy Blood and Tissue Kit (Qiagen, Hilden, Germany) following the manufacturer’s instructions. Genomic tick DNA was extracted from 100 μl of individually crushed tick material using the Wizard genomic DNA purification kit (Promega, Charbonnières-les-Bains, France) as previously described [27]. DNA was stored at -20 °C until further use.

#### Real-time PCR

A real-time PCR assay was performed on DNA extracts from blood (*n* = 550) and ticks (*n* = 355) using the primers and probe (FAM and BHQ1 as reporter and quencher molecules, respectively) (Table 1) targeting the *msp4* gene for *A. ovis* [31]. Real-time Taqman PCR was performed in a final volume of 12 μl using LightCycler® 480 Probe Master Mix (Roche Applied Science, Penzberg, Germany) at 1× final concentration. Primers and probe had a concentration of 200 nM, and each reaction included 2 μl of DNA. Negative (water) and positive controls (diluted plasmid, recombinant pBlue scriptIISK+, containing the target gene *msp4* of *A. ovis*) were used with each run. Thermal conditions were as follows: 95 °C for 5 min, 45 cycles at 95 °C for 10 s and then 60 °C for 15 s. The program included a final cooling step at 40 °C for 10 s. Samples were considered positive at a cycle threshold (Ct) value of < 40 and characteristic amplification curves.

**Table 1:** Primers used in this study

Target gene	Primer/probe (5'-3')	Type of PCR	Tm (°C)^a^	Fragment length (bp)
*msp4*	TCATTCGACATGCGTGAGTCA	Real-time	60	92
	TTTGCTGGCGCACTCACATC		60	
	FAM-AGCAGAGAGACCTCGTATGTTAGAGGC-BQ1		70	
*gltA*	GCCGACTTTGTTGCCACTGT	Qualitative	58.7	760
	TCCAACCGCCCTTAGCACAA		59.4	
*msp4*	CCCAGCGTTTCCCTCTGTTA		57.1	497
	GAGTCCGTGGTAGAACCCAC		57.3	

^a^Melting temperature

### Analysis of genetic diversity of *Anaplasma ovis*

#### *gltA* and *msp4* primers design and qualitative PCR

*gltA* and *msp4* primers were designed in the present study using sequences available on GenBank. Sequences were aligned using MAFFT and conserved regions identified [32]. Primers targeting the conserved regions of the genes were then designed by Primer-BLAST [33]. Primer properties were further analysed using OligoAnalyzer [34]. Selected primers were then synthetized (Eurofins Scientific Laboratories, Paris, France) (Table 1). Amplifications were achieved with Phusion High-Fidelity DNA Polymerase (Thermo Fisher Scientific, Waltham, MA, USA) and a Doppio thermocycler (VWR, Radnor, PA, USA). For each reaction, 4 μl of buffer solution, 0.4 μl of dNTPs (10 mM), 1 μl of forward and reverse primer (20 μM), 0.2 μl of DNA Phusion polymerase (0.02 U/μl) and 5 μl of genomic DNA were used in a final volume of 20 μl.

#### Selection of samples for sequencing

We attempted to amplify fragments of *gltA* and *msp4* genes in the 286 goat samples positive to *A. ovis* by real-time PCR. No tick sample was selected for sequencing. Amplicons were deposited on 1.5% agarose gel stained with BET and migrated in TAE 1× to check their length using a 1 kb DNA Ladder (NEB, Hitchin, UK). When a single band at the expected length was observed, the PCR product was considered positive and kept at -20 °C until sequencing. If in addition to the band of the expected size, several bands were observed, the band of the expected size was excised from the gel and purified using GeneJET Gel Extraction Kit (Thermo Scientific). The 143 and 142 samples positive for *gltA* and *msp4* were sent for sequencing and 133 and 103 sequences, respectively, were obtained.

#### Sequence alignment and phylogenetic analysis

*gltA* homologous sequences were retrieved from GenBank to represent different species of *Anaplasmataceae*, genera *Anaplasma*, *Ehrlichia* and *Neorickettsia*, as follows: *Anaplasma ovis* (GenBank: KJ410285), *A. marginale* (AF304139), *A. centrale* (AF304141), *A. capra* (KM206274), *A. platys* (AB058782), *A. phagocytophilum* (AY464138), *A. bovis* (KU586317), *Ehrlichia ruminantium* (DO513397), *E. ewingii* (DQ365879), *E. canis* (AY647155), *E. chaffeensis* (AF304142), *E. muris* (AF304144), *Neorickettsia sennetsu* (AF304148) and *N. risticii* (AF304147). For *msp4*, homologous sequences of different species belonging to the genus *Anaplasma* were retrieved from GenBank as follows: *A. ovis* (KU497712), *A. marginale* (KU497715), *A. centrale* (AF428090), *A. capra* (KM206277) and *A. phagocytophilum* (EU008082). Sequences were translated into proteins using the ExPASy translate tool [35]. For strain analysis, *msp4* nucleotide and MSP4 protein sequences belonging to *A. ovis* strains from all over the world were selected from GenBank as shown in Additional file 1: Figure S1 and Additional file 2: Figure S2, respectively. For each gene, sequences were aligned with MAFFT (v.7) configured for the highest accuracy using the scoring matrix 200PAM/kD2, alignment strategy MAFFT-FFT-NS-I, gap opening penalty 1.53 and offset value 0.123.

Non-aligned regions were removed using Molecular Evolutionary Genetics Analysis (MEGA) v.6 software [36]. The best-fit model of sequence evolution was selected based on the corrected Akaike information criterion (cAIC) and Bayesian information criterion (BIC) implemented in MEGA. Tamura 3-parameters [37] and JTT models [38], which had the lowest values of cAIC and BIC, were chosen for *gltA* and *msp4* and MSP4 tree reconstruction, respectively. The maximum likelihood method, implemented in MEGA, was used to obtain the best tree topologies. Reliability of internal branches was assessed using the bootstrapping method with 1000 bootstrap replicates [39].

### Statistical analysis

The individual/flock prevalence was calculated as the ratio of positive animals/flocks on sampled animals/flocks, a positive flock being a flock with at least one positive animal. Within-flock prevalence was calculated as the ratio of positive animals on sampled animals in a given flock. Relationships between qualitative results, correlations between quantitative variables and comparisons of means were tested with Chi-square or Fisher’s tests, Spearman’s rank correlation test and Kruskal-Wallis test using R software v.3.1.0 [40]. Alpha was set at 0.05.

A principal components analysis (PCA) was performed with R Studio [41] using the following matrix: the rows were the 55 flocks and columns were the corresponding values for *A. ovis* infection (number of infected animals), paratuberculosis seropositivity (number of seropositive animals), gastrointestinal strongyles egg excretion (pooled faecal egg count) and anaemia prevalence (number of anaemic animals). The variables were represented on the principal plane. The nearer the variables were located to the correlation circle, the better they were represented in the plane. The relative position of the variables on the plane corresponded to either a positive (near variables), a negative (variables diagonally opposed on the plane), or no association (variables at a right angle).

## Results

### General and health data

The 55 dairy goat flocks were distributed on the whole area of the island, except for the central mountain chain, at a mean altitude of 329 m (range: 19–810 m) (Fig. 1). The mean number of goats per flock was 148 (range: 27–500) and the breed was mainly the Corsican breed (76% of flocks), remaining breeds being Alpine and crossbred. At the individual level, the mean haematocrit was 27.6 ± 5.0% (range: 16.5–41.4%) with 23% of goats exhibiting anaemia (Ht ≤ 24%). At the flock level, 78% of flocks showed at least one goat with anaemia and the mean within-flock prevalence of anaemia was 23 ± 22% (range: 0–100%). The individual seroprevalence of PTB was 9.8% (excluding 27 vaccinated animals) whereas the within-flock seroprevalence varied from 0 to 50% and was 9.4% on average. Pooled faecal egg counts in the flocks ranged between 0–4500 epg with an average of 517 epg. Excretion was < 500, 500–2000 and > 2000 epg in 74.5, 20 and 5.5% of the farms, respectively.

### Prevalence of *Anaplasma ovis* DNA in goats

**Fig. 2:**
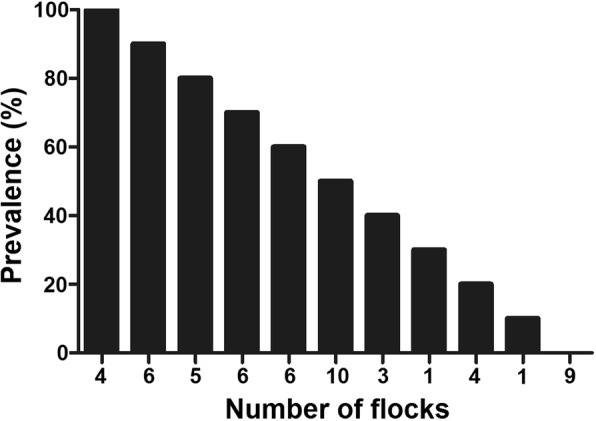
*Anaplasma ovis* prevalence in 55 goat flocks of Corsica. The prevalence of *A. ovis* detected by real-time PCR was highly variable among herds; while some herds had 0% infection rate, others had 100% infection rate

The individual prevalence of *A. ovis* DNA in goats was 52.0%, including 286 positive samples on 550 total samples. The within-flock prevalence (55 flocks) estimated from 10 individual results per flock varied from 0 to 100% with a mean of 52 ± 32% (Fig. 2). Fourteen and 15 herds had infection rates below 20% and above 80%, respectively. The *A. ovis* flock prevalence was 83.6% (flocks showing at least one *A. ovis* positive animal). Individual prevalence was significantly higher at an altitude > 168 m, in the southern county, in animals of Corsican breed and in animals aged > 3 years (Table 2). Similar associations were observed for flock prevalence with altitude and breeds.

**Table 2:** Individual prevalence of *Anaplasma ovis* in dairy goat in Corsica according to altitude, county, goat breed and age

	Altitude in m (terciles)			County		Goat breed		Goat age	
	0–168	168–437	> 437	Corse du Sud	Haute-Corse	Corsican + cross bred	Other	≤ 3 years	> 3 years
Individual prevalence (%)	36.7	53.3	65.3	60.9	45.6	59.5	27.7	42.9	62.1
*P-*values of Chi-square test	*P* < 0.05			*P* < 0.05		*P* < 0.05		*P* < 0.05	

### Relationship between *Anaplasma ovis* infection and health parameters at individual or flock levels

**Fig. 3:**
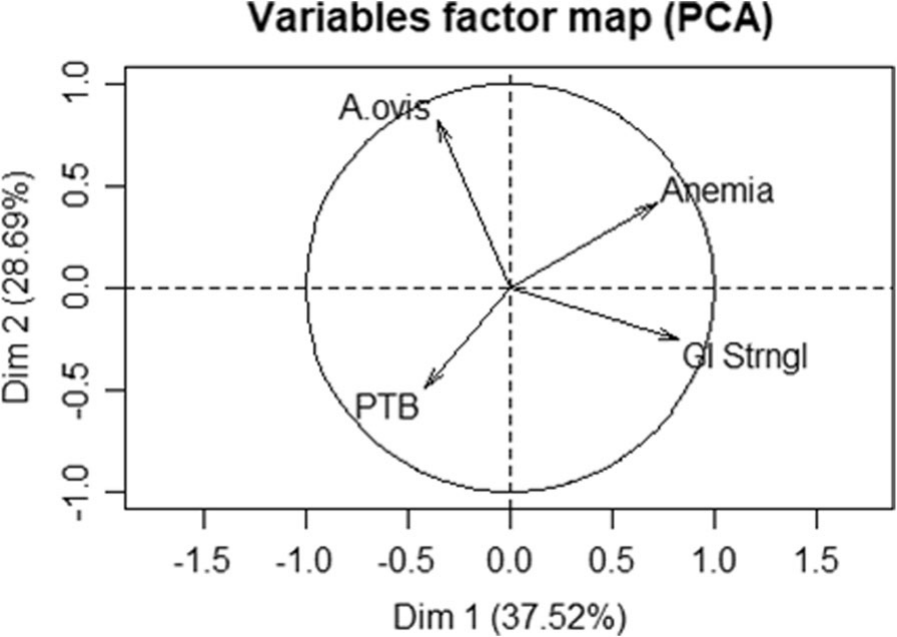
Principal components analysis to test relation between *Anaplasma ovis* infection, anaemia and other health indicators. Relationships between number of *A. ovis* infected animals (‘*A. ovis*’), number of paratuberculosis seropositive animals (‘PTB’), gastrointestinal strongyles pooled faecal egg count (‘GI Strngl’) and number of anaemic animals (‘Anemia’) in 55 goat flocks of Corsica. Principal components analysis (PCA) variables are located on the main plane defined by the 1st and 2nd axis and circle of correlation

The individual prevalence of *A. ovis* DNA was not different between goats with or without anaemia (55.9 *vs* 50.8%, *P* = 0.31). Furthermore, no significant correlation was seen at flock level between within-flock prevalence of *A. ovis* DNA and within-flock prevalence of anaemia (*r*_*s*_ = 0.14, *P* > 0.1). Results of the principal components analysis using *A. ovis* DNA detection, GIN egg excretion, PTB seropositivity and anaemia prevalence at flock level as variables are presented in Fig. 3. The first two axes explained 66% of the total inertia and the main contributors were GIN and anaemia on 1st axis and *A. ovis* on the 2nd axis. *Anaplasma ovis* was unrelated to anaemia, PTB seropositivity and GIN. However, a close association was found between GIN and anaemia (particularly on the 1st axis).

### Tick burden and prevalence of *Anaplasma ovis* infection in ticks

Ticks were found in the whole area of the survey. Zero to 27 ticks were observed from each goat and at least one tick was recorded on a goat in 58.2% of flocks. All sampled specimens (*n* = 355) were *Rhipicephalus bursa* adult ticks. No significant relationship was observed between *A. ovis* within-flock prevalence and the number of ticks observed in the flocks (*P* = 0.46). *Anaplasma ovis* DNA was detected in 20.3% of the ticks and 51.6% of the tick-infested flocks showed at least one *A. ovis*-infected tick. Tick positive samples were not selected for sequencing.

### *Anaplasma ovis gltA* and *msp4* nucleotide sequences and phylogenetic analysis

From the 286 positive goat samples, we attempted to amplify fragments of *gltA* and *msp4* genes for further sequence and phylogenetic analysis. Using the primers shown in Table 1 for a qualitative PCR assay, 143 (50.0%) and 142 (49.7%) samples were positive for *gltA* and *msp4*, respectively. Among these positive samples, 86 (30.8%) and 99 (35.4%) were positive for one of the genes (*gltA* or *msp4*) and the two genes (*gltA* and *msp4*), respectively. From the 143 *gltA-*positive and 142 *msp4-*positive samples, 133 (93.0%) and 103 (72.5%) sequences, respectively, were obtained.

**Fig. 4:**
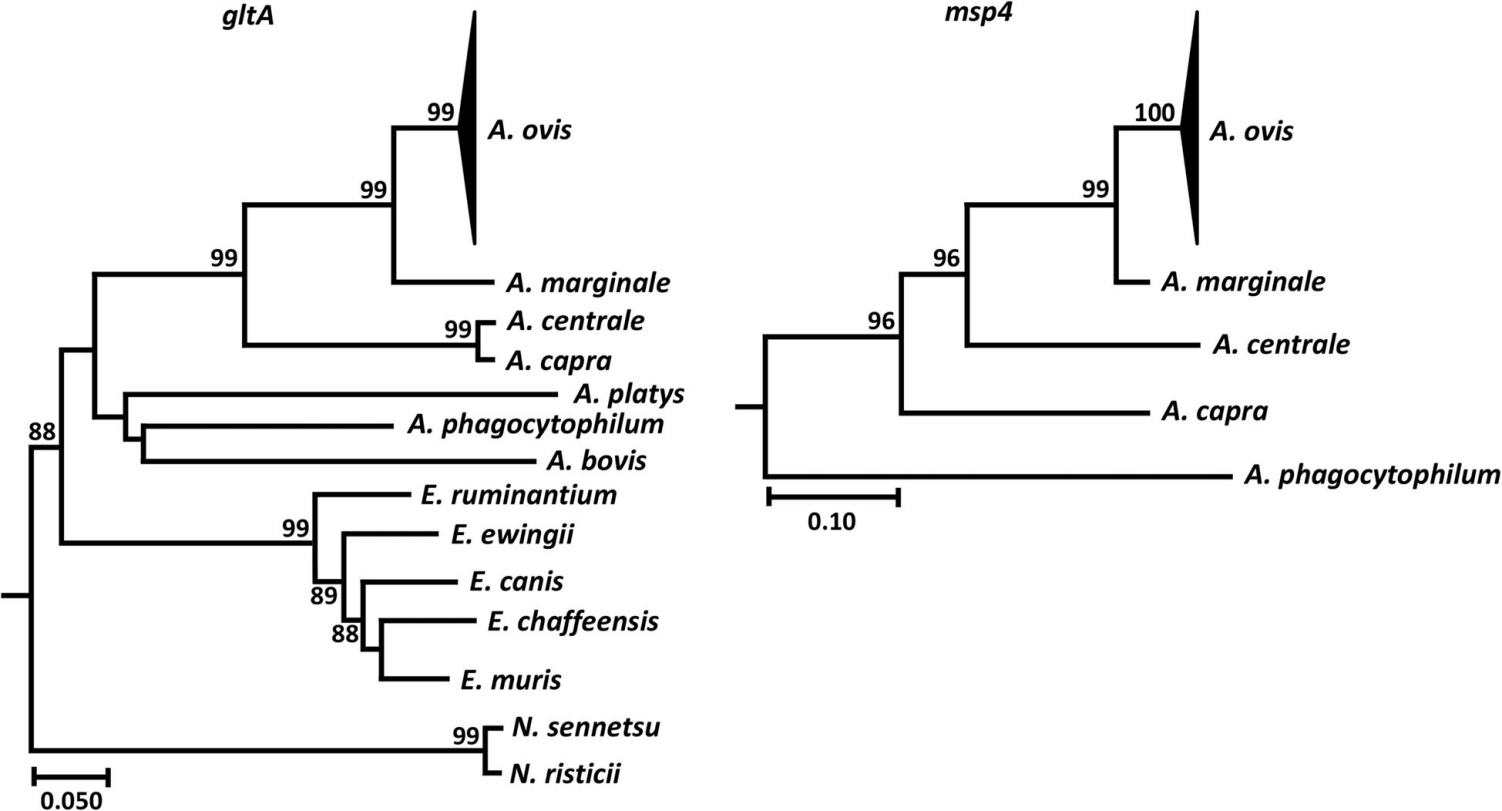
Phylogenetic analysis of *Anaplasma ovis gltA* and *msp4* nucleotide sequences identified in Corsica. Maximum likelihood phylogenetic trees were inferred using *gltA* and *msp4* nucleotide sequences of *A. ovis* identified in Corsica and other bacteria of the family *Anaplasmataceae* (see Methods for accession numbers). All *gltA* and *msp4* nucleotide sequences of Corsica formed a unique clade with *A. ovis* reference sequences, KJ410285 (*gltA*) and KU497712 (*msp4*). The clades containing *A. ovis* sequences were collapsed. Reliability of internal branches was assessed using the bootstrapping method (1000 replicates)

Species classification based on real-time and qualitative PCR assays was confirmed by sequencing. All amplified *gltA* sequences formed a unique clade with an *A. ovis* reference sequence (GenBank: KJ410285) included in the phylogenetic tree (Fig. 4). Similar results were obtained when *msp4* sequences were used to build the phylogenetic tree. All amplified *msp4* sequences clustered with the *A. ovis* reference sequence (GenBank: KU497712) (Fig. 4).

### *Anaplasma ovis* strain diversity analysis using MSP4 sequences

**Fig. 5:**
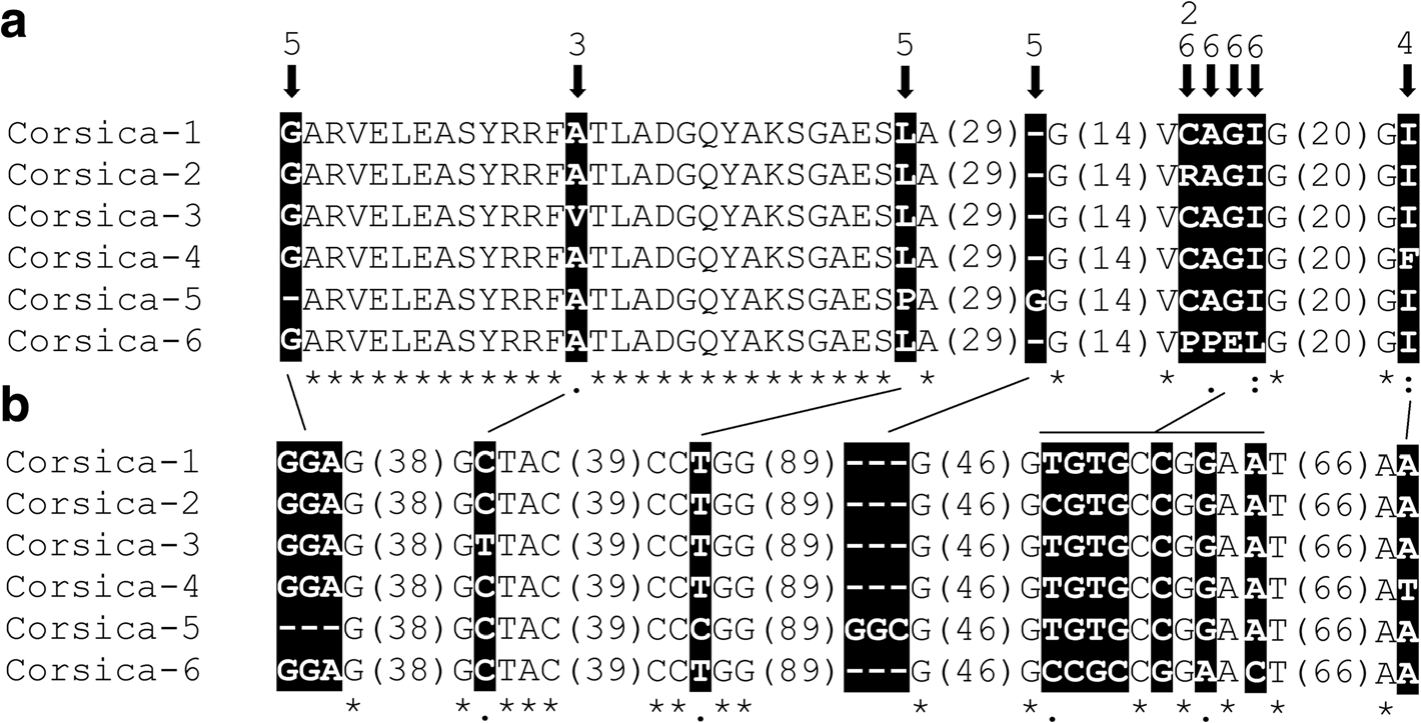
*Anaplasma ovis* strain analysis based in *msp4* sequences. **a** Six different strains of *A. ovis* were identified in Corsica (COR 1-6, GenBank: MH121148-MH121152) and positions with amino acid polymorphisms among them are highlighted in black boxes. For figure simplification purpose, large stretches of regions with 100% identity were removed from the figure and the amino acid length of these regions is shown in parentheses. **b** The non-synonymous nucleotide substitutions and indels resulting in MSP4 amino acid variability are highlighted in black boxes

Following theoretical translation of all *msp4* nucleotide sequences into proteins, six different sequences were identified and named strains Corsica 1-6 (hereafter COR1-6, GenBank: MH121148-MH121152). Protein sequence alignment revealed amino acid substitutions and indels that explained the genetic diversity among these strains (Fig. 5a). The strain COR1 was the most frequently found and therefore it was considered as the reference strain in further comparisons. COR2 differed from the reference sequence in a substitution from cysteine (C) to arginine (R). Compared to COR1, strains COR3 and COR4 also had one amino acid substitution from alanine (A) to valine (V) and from isoleucine (I) to phenylalanine (P), respectively. Remarkably, strain COR5 had one deletion and one insertion of a glycine (G) compared to COR1-4 and COR6 and, in addition, a substitution from leucine (L) to proline (P) (Fig. 5a). Finally, strain COR6 differs from the reference sequence by a fragment of 4 consecutive amino acids, specifically PP - glutamic acid (E) - L (PPEL) to CAGI. Except for strains COR2 and COR6 with one amino acid change in a common position, amino acid substitutions were located in different amino acid positions along the MSP4 sequence. The change of hydrophilic into hydrophobic amino acid, and *vice versa*, was observed twice, one in strain COR2 (C to R) and the other in strain COR6 (G to E). The other amino acid changes were conservative and resulted in hydrophobic amino acid substitutions.

### Genetic basis of *msp4* sequence variability

Nucleotide sequences analysis of *msp4* revealed non-synonymous substitutions that resulted in the MSP4 amino acid variability (Fig. 5b). Strains COR2 and COR5 differed from the reference strain in one nucleotide substitution, thymidine (T) to cytosine (C). In addition, compared to COR1, strain COR3 had T in place of C and strain COR4 contained T instead of adenine (A). Finally, 7 nucleotide substitutions were identified in strain COR6 compared to COR1. In particular, COR6 had 4 successive substitutions (CCGC instead of TGTG; G corresponding to guanine) and 3 additional substitutions as follows: C to G, G to A and A to C. Only two synonymous substitutions were identified, one in the first aspartic acid (D) codon of strain COR3 (C to T) and the other in the second V codon of strain COR4 (T to G).

### Frequency of *Anaplasma ovis* strains in goats in Corsica

The strain COR1 was the most represented and was found in 95 samples, corresponding to 94.6% of the *msp4* sequences obtained. Strain COR2 was found in two samples and represented 1.9% of the sequences obtained. The strains COR3-6 were represented by only 1 sample and each corresponded to 0.9% of the sequences obtained.

### Comparison between *Anaplasma ovis* strains in Corsica and other regions

**Fig. 6:**
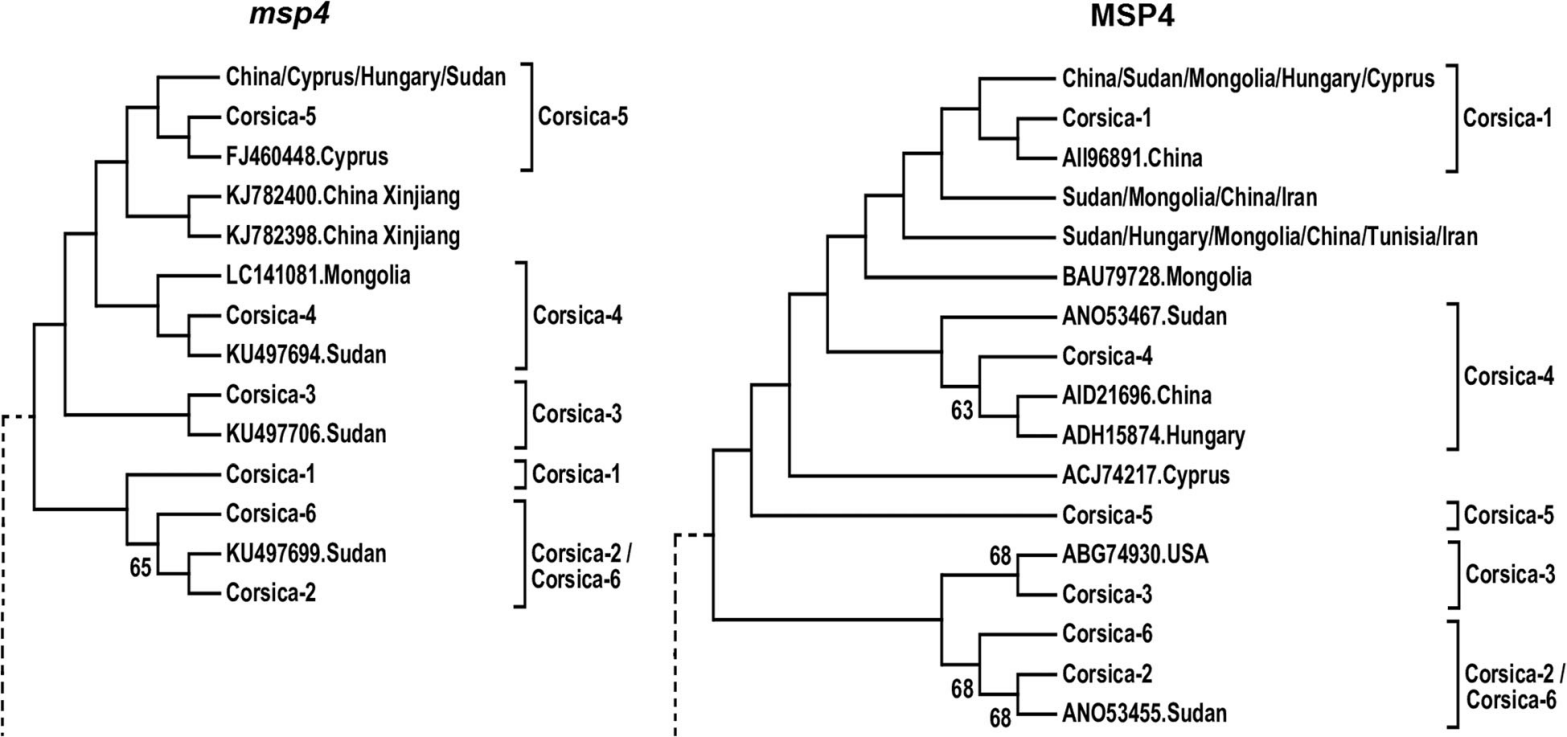
Phylogenetic analysis of *Anaplasma ovis msp4* sequences identified in Corsica and other regions of the world. Maximum likelihood phylogenetic trees were inferred using *msp4* nucleotide and MSP4 protein sequences of *A. ovis* of Corsica (COR 1-6, GenBank: MH121148-MH121152) and other regions of the world. To simplify figure display, clades that did not contain Corsica strains were removed (dashed lines) or collapsed (countries of origin in bold letters). Clades containing different Corsica strains were labeled with the name of the strain. A detailed version of the cladograms for *msp4* and MSP4 are available in Additional file 1: Figure S1 and Additional file 2: Figure S2, respectively. GenBank accession numbers and country of origin are shown

Among the six *A. ovis* strains identified in Corsica, the *msp4* nucleotide and MSP4 protein sequences of COR1 had 100% identity with strains previously reported in Cyprus (GenBank gene/protein accession numbers FJ460454/ACJ74223), Mongolia (GenBank gene/protein accession numbers LC141078/BAU79725) and other countries. None of the other strains (COR2, COR3, COR4, COR5 and COR6) were 100% identical to any previously reported *A. ovis* strains. To study the evolutionary history of the *A. ovis* strains of Corsica, phylogenetic trees were constructed using *msp4* nucleotide and MSP4 protein sequences. Sequences that were 100% identical between them were excluded from the analysis. Except for COR2 and COR6 that were closely related between them (Fig. 6), both trees showed that the other COR strains fall in independent clusters.

## Discussion

Using a large sample (*n* = 550) and molecular tools, we made a robust assessment of the prevalence, distribution and genetic diversity of *A. ovis* in goats in Corsica. The main findings of this study were: (i) high prevalence and wide distribution of *A. ovis* in Corsica; (ii) no link between health indicators and *A. ovis* infection in goats in apparent healthy flocks; and (iii) high genetic diversity of *A. ovis*. Our results are the first report of *A. ovis* in goats from Corsica and should be compared to the prevalence reported in other Mediterranean areas. *Anaplasma ovis* has been reported in Sicily [42], Sardinia [43] and Cyprus [44]. Corsica, Sicily, Sardinia and Cyprus share similar ecosystems, providing good models of the epidemiological traits of *A. ovis* in the Mediterranean basin and showing large variations in *A. ovis* prevalence from one area to another. For instance, in Italy, *A. ovis* prevalence in goats was 31.7% in southern Italy [20], 19% in Sicily [42] and 81.8% in Sardinia [43]. It is noteworthy that *A. ovis* was previously reported in sheep in Corsica [45] and Italy [20, 42, 43].

In our study, goats aged over three years, of Corsican breed/crossbred and raised above 168 m of altitude were more at risk for *A. ovis* infection. Infection of aged animals was previously reported by Zhou et al. [46] in sheep in Turkey and could be explained by potentially higher contact rates with infected vectors in the field. In contrast, the breed and altitude effects remain unclear because breeds other than Corsican/crossbred are mainly raised on the lowlands. Aktas & Ozübeck [47] reported an association between *A. ovis* infection in sheep and goats and the presence of *Rhipicephalus bursa* and *R. turanicus* in four provinces of south-eastern Turkey. In our study, *R. bursa* was the only tick species found on goats, which agrees with the study of Grech-Angelini et al. [25] who reported this tick species as being the most abundant on goats in Corsica. *Rhipicephalus bursa* is a typical tick species of the Mediterranean area from coast to mountainous area and has been shown to be one of the main biological vectors of *A. ovis* both in natural and experimental conditions [1]. We found 20.3% of *R. bursa* infected with *A. ovis* which can be considered as a high rate of infection when compared with 7.9% in a previous survey in the French Basque Country after an epizootic outbreak of sheep anaplasmosis [48]. These results suggest a high level of transmission of *A. ovis* between ticks and goats in Corsica.

Most of published studies on *A. ovis* prevalence were performed on clinically healthy sheep or goats. However, as for other *Anaplasma* spp., *A. ovis* infection could be more severe in stressful situations or in the presence of co-infections [6, 15]. In this study, we investigated the relationship between *A. ovis* infection at individual and flock level and some heath parameters (i.e. haematocrit, GIN egg excretion and paratuberculosis seropositivity). No significant bivariate or multivariate association was found between these health indicators suggesting that *A. ovis* infection does not affect these parameters. Although haematocrit is considered as one of the most informative clinical criterion for *A. ovis* infection in sheep [49], this parameter is not specific and this was illustrated in our study with the association between anemia and GIN egg excretion through principal components analysis. Regarding tick-borne pathogen co-infection, Aktas & Ozübeck [47] showed that sheep infected with *Babesia* and *Theileria* had higher *A. ovis* prevalence in Turkey [47]. The possible occurrence of co-infections in goats deserves special attention because *R. bursa* is the vector of many important tick-borne pathogens occurring in livestock in Corsica [25, 45, 50].

The *msp4* gene is routinely used for the characterization of genetic diversity of *Anaplasma* spp., including *A. marginale*, *A. phagocytophilum* and *A. ovis* [17, 51]. Based on *msp4* sequence analysis, we found a relatively high genetic diversity of *A. ovis* in Corsica where six strains (COR1-6) were identified. Two lines of evidence supported the idea of high genetic diversity of *A. ovis* in Corsica. First, based on the phylogenetic analysis, except for COR2 and COR6, the six *A. ovis* COR strains fell in different clusters (Fig. 6). This suggests that most COR strains do not share a recent common ancestor. Secondly, compared with previous reports in different host species and regions [13, 52–54], six is a relatively high number of *A. ovis* strains identified in a relatively small area such as Corsica. For instance, in Turkey, only one *A. ovis msp4* genotype was reported in goat and sheep [46]. A similar result was reported in Sicily [42, 55]. In Tunisia, only two *A. ovis msp4* genotypes, previously described in Italy, were identified in sheep and goats [54]. Low genetic diversity was also reported in North America where only one *msp4* genotype was found in Bighorn sheep [17]. Likewise, only one *A. ovis msp4* genotype, closely related to the *A. ovis* genotype AOI from Italy, was found in sheep in the region of Xinjiang, northwest China [21]. Another study conducted to investigate the occurrence and characterize *A. ovis* strains from goats and sheep from 12 provinces in China identified only two *msp4* genotypes of these bacteria [13]. Thus, genetic diversity analysis of *A. ovis msp4* rarely identified more than two genotypes in sheep or goats. In contrast to the above reports, and in agreement with the results of this study, one study in continental Europe (i.e. Hungary) reported five *A. ovis msp4* variants in samples collected from sheep across the whole country [52]. In addition, phylogenetic analysis of *A. ovis msp4* sequences from sheep and goats in Hubei, Guizhou, Zhejiang and northern China identified six different genotypes [56]. Considering the size of the studied regions, it is remarkable that the same number of *A. ovis* strains (i.e. six) was found in this study in Corsica (area 8 680 km^2^) and China (area 9 597 000 km^2^) [56].

While the genetic diversity of *A. ovis* between regions is variable, it is a common finding of studies addressing the genetic diversity and prevalence of *A. ovis* that one *A. ovis* strain is frequently found to be dominant (i.e. infecting most of the animals) [13, 52–54]. In agreement with previous reports, the strain COR1 was found in 94.6% of the *msp4* sequences obtained, while the other strains (COR2-6) were poorly represented. However, the association between high genetic diversity and high prevalence of *A. ovis* in Corsica differs from previous reports. For instance, low genetic diversity of *msp4* sequences was associated with low and high prevalence of *A. ovis* in China [13] and Turkey [17, 53], respectively. In the study by Liu et al. [56], low prevalence (15.3%) was associated with high genetic diversity. Thus, the epidemiological setting of *A. ovis* in Corsica can be considered as unique compared to that reported in China and Turkey.

## Conclusions

In this study, we detected a high individual and flock prevalence of *A. ovis* DNA in clinically healthy goat flocks in Corsica and the occurrence of *A. ovis* DNA in *Rhipicephalus bursa* ticks. No relationship was found between *A. ovis* infection at the individual or flock level and some relevant health indicators such as haematocrit, gastrointestinal infection or paratuberculosis seropositivity. The high prevalence of *A. ovis* in goats in Corsica was associated with high genetic diversity of this bacterium, one out of the six identified strains representing a large majority of the sequenced samples. Further studies are needed to evaluate the pathogenic potential of these strains for goats in this insular environment.

## Additional files

Additional file 1: **Figure S1**. Phylogenetic analysis of *Anaplasma ovis msp4* sequences identified in Corsica and other regions of the world. The figure shows a maximum likelihood phylogenetic tree inferred using *msp4* nucleotide sequences of *A. ovis* of Corsica (Corsica 1-6) and other regions of the world. GenBank accession numbers and country of origin are shown. (PDF 15 kb)

Additional file 2: **Figure S2**. Phylogenetic analysis of *Anaplasma ovis* MSP4 sequences identified in Corsica and other regions of the world. The figure shows a maximum likelihood phylogenetic tree inferred using MSP4 amino acid sequences of *A. ovis* of Corsica (Corsica 1-6) and other regions of the world. GenBank accession numbers and country of origin are shown. (PDF 13 kb)

## Abbreviations

PCR: Polymerase chain reaction
qPCR: Quantitative PCR
dNTPs: Deoxynucleotide triphosphates
GIN: Gastrointestinal nematodes
PTB: Paratuberculosis
msp4: Major surface protein 4
*gltA*: Citrate synthase
DNA: Deoxyribonucleic acid
Ht: Hematocrit
FEC: Faecal egg count
epg: Eggs per gram
PCA: Principal components analysis
