# Supplementary figures and images for: Epidemiology and genetic diversity of Anaplasma ovis in goats in Corsica, France
Source: Parasit Vectors. 2019 Jan 3;12:3. doi: 10.1186/s13071-018-3269-7 (PMC6318933; doi:10.1186/s13071-018-3269-7)

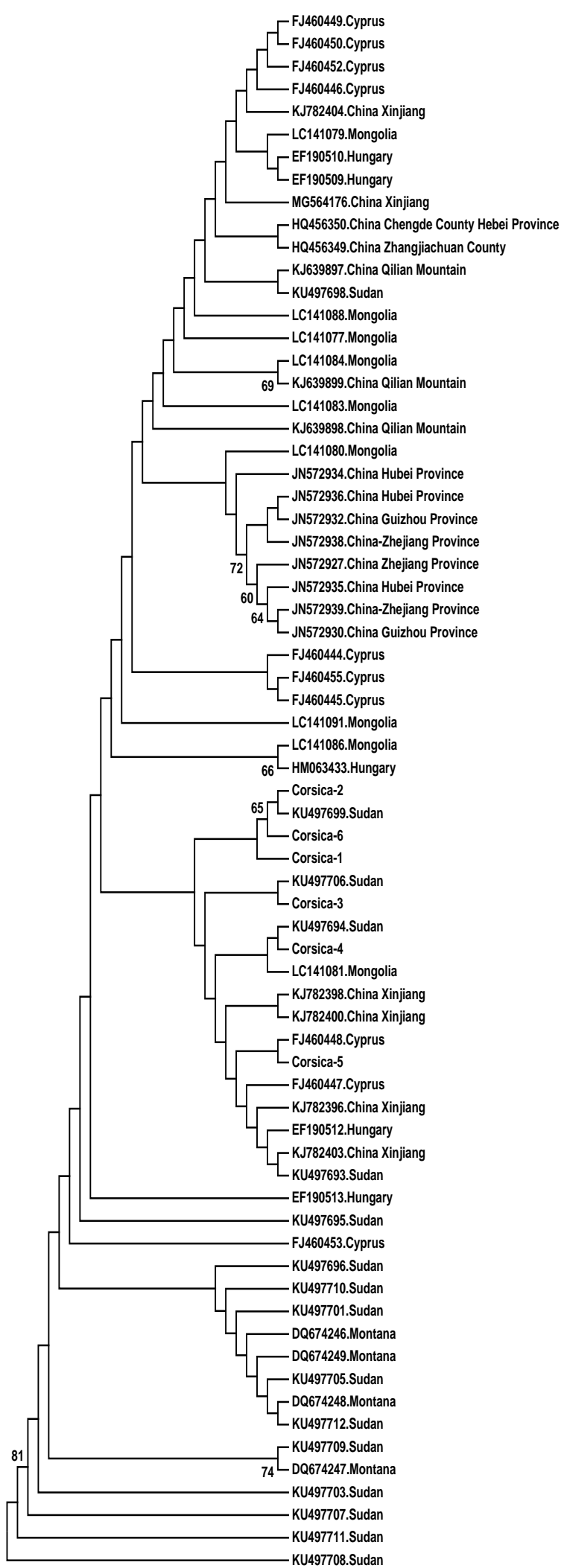

Supplement: Supplementary file 1 — Figure S1. Phylogenetic analysis of Anaplasma ovis msp4 sequences identified in Corsica and other regions of the world. The figure shows a maximum likelihood phylogenetic tree inferred using msp4 nucleotide sequences of A. ovis of Corsica (Corsica 1-6) and other regions of the world. GenBank accession numbers and country of origin are shown. (PDF 15 kb) [file 13071_2018_3269_MOESM1_ESM.pdf]

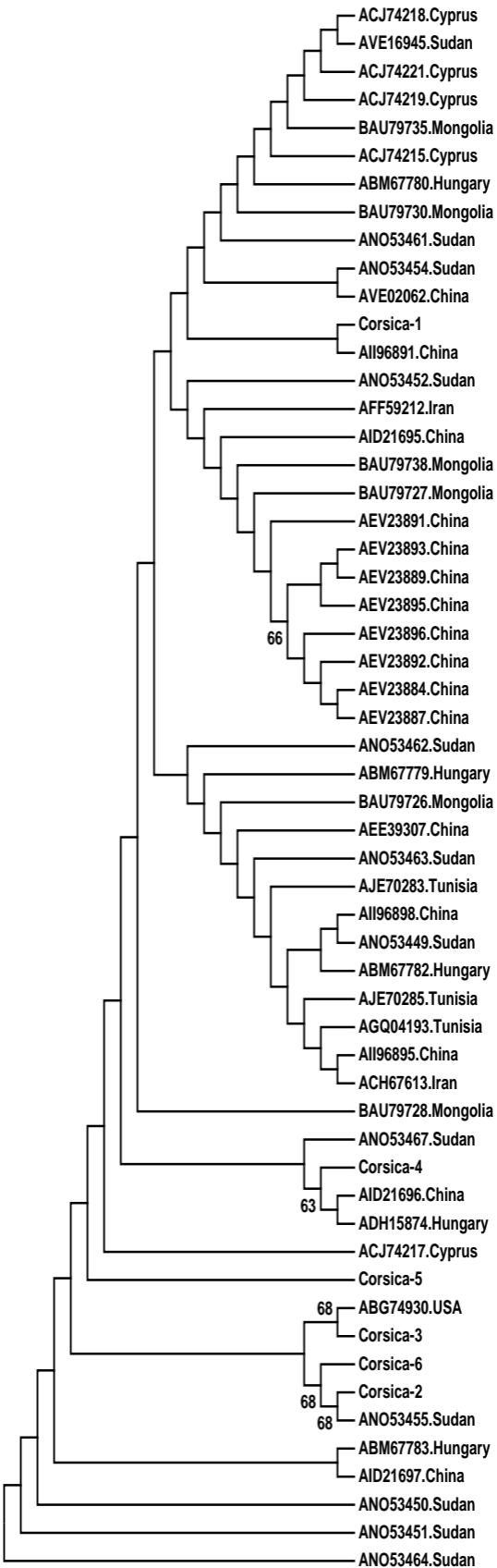

Supplement: Supplementary file 2 — Figure S2. Phylogenetic analysis of Anaplasma ovis MSP4 sequences identified in Corsica and other regions of the world. The figure shows a maximum likelihood phylogenetic tree inferred using MSP4 amino acid sequences of A. ovis of Corsica (Corsica 1-6) and other regions of the world. GenBank accession numbers and country of origin are shown. (PDF 13 kb) [file 13071_2018_3269_MOESM2_ESM.pdf]
